# Study on the synergistic protective effect of *Lycium barbarum* L. polysaccharides and zinc sulfate on chronic alcoholic liver injury in rats

**DOI:** 10.1002/fsn3.1182

**Published:** 2019-09-12

**Authors:** Yamei Yan, Wanqiang Wu, Lu Lu, Jie Ren, Jia Mi, Xuebo Liu, Youlong Cao

**Affiliations:** ^1^ National Wolfberry Engineering Research Center Yinchuan China; ^2^ College of Food Science and Engineering Northwest A&F University Yangling China

**Keywords:** alcoholic liver injury, *Lycium barbarum* L., polysaccharides, synergistic effects, ZnSO_4_

## Abstract

Both *Lycium barbarum* L. polysaccharides (LBP) and zinc have protective effects on liver injuries. In this paper, LBP and ZnSO_4_ were combined to study the effects on the prevention of alcoholic liver injury. The rats were divided into six groups, the normal group, alcohol group, zinc sulfate group, LBP group, low‐dose group of ZnSO_4_, and high‐dose group of ZnSO_4_ and LBP, used to explore the impact of LBP and ZnSO_4_ complex on liver lipid metabolism of alcohol, alcohol‐metabolizing enzymes, oxidative damage, and inflammation of the liver. The experimental model was established by gavage treatment, observation, and determination of indexes of rats. The results showed that the combination of LBP and ZnSO_4_ could significantly decrease the levels of triglyceride (TG), total cholesterol (TC), tumor necrosis factor‐α(TNF‐ɑ), malondialdehyde (MDA), alanine aminotransferase (ALT), aspartate aminotransferase (AST), and the activity of enzyme subtype 2E1 (CYP2E1). It also significantly increased the activities of total superoxide dismutase (SOD), catalase (CAT), glutathione peroxidase (GSH‐Px), glutathione peptide (GSH), and alcohol dehydrogenase, effectively improved the liver tissue lesion. What is more, the combination of LBP and ZnSO_4_ had a synergistic effect on the remission of alcoholic fatty liver, and alleviated chronic alcoholic liver injury by promoting lipid metabolism, inhibiting oxidative stress, controlling inflammatory responses, and regulating the expression and activity of alcohol‐metabolizing enzymes in rats.

## 1 INTRODUCTION

Alcoholic liver disease (ALD) is due to excessive alcohol intake caused by liver damage and a series of lesions; the pathogenesis is more complex, making it become a worldwide medical problem (Corrao, Bagnardi, Zambon, & LaVecchia, 2004), which due to a high morbidity and mortality worldwide (Chiang & McCullough, 2014). Much data on the pathogenesis of ALD have been obtained from animal studies (Altamirano & Bataller, 2011; Ceni, Mello, & Galli, 2014; Gao & Bataller, 2011; Orman, Odena, & Bataller, 2013). The main reason is that acetaldehyde, an intermediate alcohol metabolite, can deplete glutathione, accelerate lipid peroxidation, mitochondria damage, lead to oxidative stress (Ceni et al., 2014; Cheng & Kong, 2011). Furthermore, alcohol‐derived reactive oxygen species (ROS) may directly trigger the systemic inflammatory response, activate nuclear factor kappa B (NF‐κB) simultaneously, which results in production of inflammatory cytokines, such as TNF‐α and IL‐6 (Cheng & Kong, 2011). Alcohol‐derived ROS may initiate a vicious cycle via the hepatocyte damage mechanism with additional inflammatory cytokines and ROS production (Ark, Lee, & Lee, 2014). Moreover, alcohol consumption increases a small intestinal bacterial overgrowth and intestinal permeability of endotoxins. The endotoxin‐mediated inflammatory signaling plays a major role in alcoholic liver fibrosis (Altamirano & Bataller, 2011). However, no treatment has been approved for patients with ALD yet, and the only recognized management strategies were alcohol cessation (Orman et al., 2013); therefore, development of novel pathophysiological‐targeted adjuvant therapies are urgently needed (Ghorbani, Hajizadeh, & Hekmatdoost, 2016).

*Lycium barbarum* L. is a traditional Chinese geoherbalism medicine (Bartosz & Anna, 2016), which can nourish liver, improve eyesight, and exhibit protective effects for liver function as recorded by the Compendium of Materia Medica. Modern medicine shows that *Lycium barbarum* L. is rich in polysaccharides, which are natural antioxidant and a hepatoprotective derivative (Masci et al., 2018). Gan et al. (2018) showed that an alleviating effect of LBPs on CCl_4_‐induced liver fibrosis in Wistar rats may be through inhibiting the TLRs/NF‐κB signaling pathway expression. Cheng Daye and Kong Hong showed LBP administration protected liver cells from the damage induced by ethanol (Cheng & Kong, 2011).

Zinc plays an important role in maintaining the stability of antioxidant enzymes and scavenging oxygen‐free radicals. It also plays a protective role in alcoholic liver injury (McClain, Vatsalya, & Cave, 2017). Approximately 30%–50% of individuals with alcohol dependency have a low zinc status because alcohol consumption decreases intestinal absorption of zinc and increases urinary excretion of zinc (Skalny, Skalnaya, Grabeklis, Skalnaya, & Tinkov, 2017). Zinc deficiency may also give rise to oxidative stress. Increased oxidative stress and oxidative stress‐induced damage have been observed in humans with a suboptimal zinc intake (Rajapakse, Curtis, & Chen, &Xu, 2017). A significant increase in the MDA levels and decrease in the GSH content and SOD activity were observed in the liver of rats fed on a zinc‐deficient diet; however, zinc supplementation resulted in a decrease in the MDA levels and increase in GSH content and SOD activity (Tupe, Tupe, & Agte, 2011; Tupe, Tupe, Tarwadi, & Agte, 2010). Additionally, zinc deficiency is linked to alcohol‐induced intestinal barrier dysfunction, as well as alveolar epithelial cell and macrophage dysfunction (Lenz et al., 2013; Zhong, Zhao, McClain, Kang, & Zhou, 2010).

As both LBP and Zn are potent antioxidants and could potentially help to protect the alcohol liver injury, we aimed to explore whether LBP‐ZnSO_4_ has a synergistic effect in alleviating the detrimental alterations induced by ethanol in rats, such as the imbalance between oxidation and antioxidants, liver injury, and abnormal hemorheology.

## 2 MATERIALS AND METHODS

### 2.1 Materials

*SD* rats were purchased from the Experimental Animal Center of Xi'an Jiaotong University, license number SCXK (Shanxi) 2013‐001. LBP was purchased from Zhejiang Genk Pharmaceutical Co., Ltd.. Food grade zinc sulfate was purchased from Shanxi Parnir Biotechnology Co., Ltd..

### 2.2 Chemicals

Alanine aminotransferase (ALT), aspartate aminotransferase (AST), total superoxide dismutase (T‐SOD), catalase (CAT), glutathione peroxidase (GSH‐Px), glutathione peptide (GSH), malondialdehyde (MDA), triglyceride (TG), total cholesterol (TC) and alcohol dehydrogenase (ADH) activity kits were provided by the Nanjing Institute of Biology (Nanjing, China). An enzyme subtype 2E1 (CYP2E1) enzyme‐linked immunoassay kit and a rat alcohol dehydrogenase (ADH) ELISA kit were purchased from Shanghai Xinle Biotechnology Co., Ltd.. A bicinchoninic acid (BCA) kit was purchased from Thermo Scientific Company. Ethylenediaminetetraacetic acid (EDTA) was purchased from Amresco Corporation of the United States. 3‐aminopropyl‐triethoxysilane (APES) was purchased from the United States of America sigma (manufactured by Nippon Polyamide Co., Ltd.). Eosin stain solution and hematoxylin dyeing liquid were purchased from Guangzhou Xinyuan pathology reagents Co., Ltd.

### 2.3 Animal treatment and modeling

Thirty‐six female *SD* rats of clean grade, weighing about 150 g were put into cages, maintained in a specific pathogen‐free environment (25 ± 4°C, 60% ~ 70% relative humidity, 12 hr light alternated), with 1 week of adaptive feeding and then divided into six groups randomly. Animal modeling was established according to the reported method. Animal modeling was established according to the reported method. The normal control group and alcohol group were given normal saline (0.5 ml 100 g/day) during the experiment, meanwhile, the medicine treatment groups (group C, D, E, and F) were given gavage (0.5 ml/100 g B.W./day) with ZnSO_4_ or LBP or different dosage of ZnSO_4_ and LBP compound solution to rats once a day, as shown in Table 1. After 1 hr, all the groups except the normal group were treated with 56% alcohol (V/V), and the first week with the dose 2 g kg^−1^ day^−1^, then increased to 8 g kg^−1^ day^−1^ for 8 weeks.

**Table 1:** Treatment groups table of experimental rats with different dosage (mg/kg B.W.)

Groups	ZnSO_4_	LBP
A (normal model group)	–	–
B (alcohol group)	–	–
C (ZnSO_4_ group)	24	–
D (LBP group)	–	500
E (ZnSO_4_ and LBP low–dose group)	12	250
F (ZnSO_4_ and LBP high–dose group)	24	500

Note

the ZnSO_4_ or LBP or ZnSO_4_ and LBP compound dissolved in water, forming solution, given gavage (0.5 ml/100 g B.W./day) to rats once a day before alcohol gavage.

### 2.4 Rats specimens collection

All rats were weighed daily and killed at the end of 9 weeks. Blood samples were centrifuged and collected at 2,000 r/min at 4°C for 15 min to obtain serum. Livers were totally excised from the rats and stored at −80°C for the subsequent experiments.

### 2.5 Determination method

#### 2.5.1 Body weight and liver coefficient

*SD* rats were weighed by everyday during experiment period. The liver coefficient was calculated as follows:

(1) $$\text{Liver}\;\text{coefficient}\left(g\;/\;100\;g\right)\;=\;W_{1}\;/\;W_{0}\;\times \;100$$

where *W*_1_ and *W*_0_ are wet liver weight and body weight, respectively.

#### 2.5.2 Liver histopathology studies

Rat livers from all groups were removed and fixed immediately in 4% neutral buffered formalin, dehydrated in gradual ethanol (30%–100%), cleaned in xylene, and embedded in paraffin. Sections were prepared and stained with hematoxylin and eosin (H&E) for photomicroscopic observation.

#### 2.5.3 Measurement of lipid levels in serum

TG and TC were measured by a phosphoglycerate oxidase‐PAP (GPO‐PAP) enzyme kit and cholesterol oxidase‐PAP (CHOD‐PAP) enzymatic kit, respectively.

#### 2.5.4 Oxidative stress parameters and inflammatory factors in rat livers

The levels of Serum transaminase (ALT and AST), MDA, SOD, CAT, GSH‐Px, GSH, and tumor necrosis factor‐α(TNF‐ɑ) were allied using the kits of Nanjing Jiancheng Bioengineering Institute.

#### 2.5.5 Determination of the activity and the contents of ADH and CYP2E1 in rat liver

The activity of ADH and CYP2E1 were analyzed by the double antibody sandwich method using commercially available kits (Shanghai Xin Le Biotechnology Co., Ltd.).

#### 2.5.6 Analysis of the interaction between LBP and zinc sulfate

The synergistic effect of the drug combination was analyzed using the drug interaction coefficient (CDI), which was calculated as follows (Liu et al., 2015):

(2) $$\text{CDI}\;=\;\frac{N_{\text{AB}}}{N_{A}\;\times \;N_{B}}$$

*N*_AB_ is the ratio of the corresponding parameters in ZnSO_4_ and LBP complex group to alcohol group; *N_A_* or *N*_B_ is the ratio of these parameters in the single group to the alcohol group. If CDI < 1, it means that there is a synergic effect between the two drugs; CDI = 1 means that there is an additive effect between the two drugs; CDI > 1 means that there is an antagonistic effect between the two drugs.

### 2.6 Statistical analysis

All statistical analysis was carried out using SPSS 21.0 software (SPSS). All data were expressed as mean ± *SD*, Duncan's multiple comparison (DMRT) was used to analyze the significant difference. A value of *p* < .05 was considered to be statistically significant.

## 3 RESULTS

### 3.1 Effect of LBP and ZnSO_4_ on body weight (BW) and liver coefficient in experimental rats

The body weight in experimental rats was shown in Table 2. At the start of the experiment there was no significant difference in body weight between groups (*p *> .05), and then increased in varying degrees after 3 weeks, compared with the normal model group (A), weight of the alcohol group (B)decreased significantly after 4 weeks (*p* < .05). Compared with the group B, the body weight of each group was significantly increased (*p* < .05) except group C. The results suggest that LBP group (D) and the complex of LBP and Zn group (E, F) can alleviate the symptom of weight loss in the model of alcoholic liver injury.

**Table 2:** Effect of LBP and ZnSO_4_ on body weight (BW) in experimental rats (g)

Groups	Alcohol	ZnSO_4_	LBP
A (normal model group)	–	–	–
B (alcohol group)	8,000	–	–
C (ZnSO_4_ group)	8,000	24	–
D (LBP group)	–	–	500
E (ZnSO_4_ and LBP low–dose group)	8,000	12	250
F (ZnSO_4_ and LBP high–dose group)	8,000	24	500

Note

The data were expressed as means ± *SD* (*n* ≥ 3)；# means compared to the control group (A), *p* < .05; *means compared to the alcohol group (B), *p* < .05.

**Figure 1:**
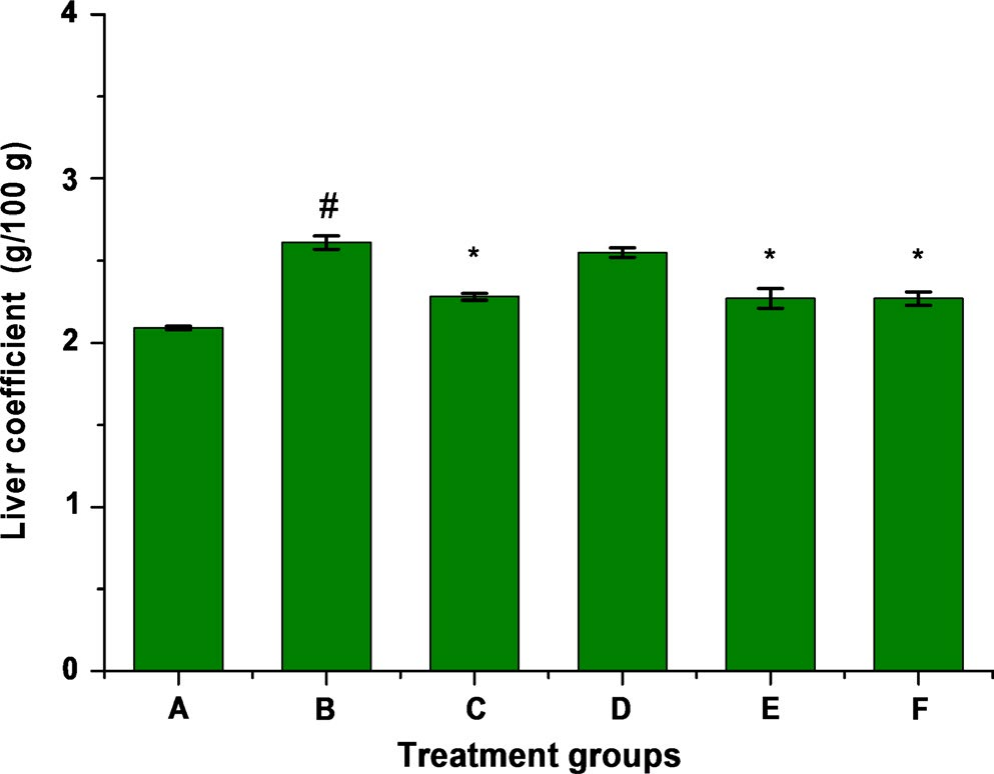
Effect of Complex solution on liver index in alcohol‐induced chronic hepatic injury. Note: The data are presented as the mean ± *SD* (*n* ≥ 3); # means compared to normal model group, *p* < .05; * means compared to alcohol group, *p* < .05

Compared the normal model group, the liver coefficient was increased significantly (*p* < .05) in alcohol group as shown in Figure 1; compared the alcohol group, the liver coefficient of group C, E, F are decreased significantly (*p* < .05) except group D, it indicated that compound of LBP and Zn can make the liver coefficient return to a normal level.

### 3.2 Effect of LBP and ZnSO_4_ on serum lipid metabolism in experimental rats

**Figure 2:**
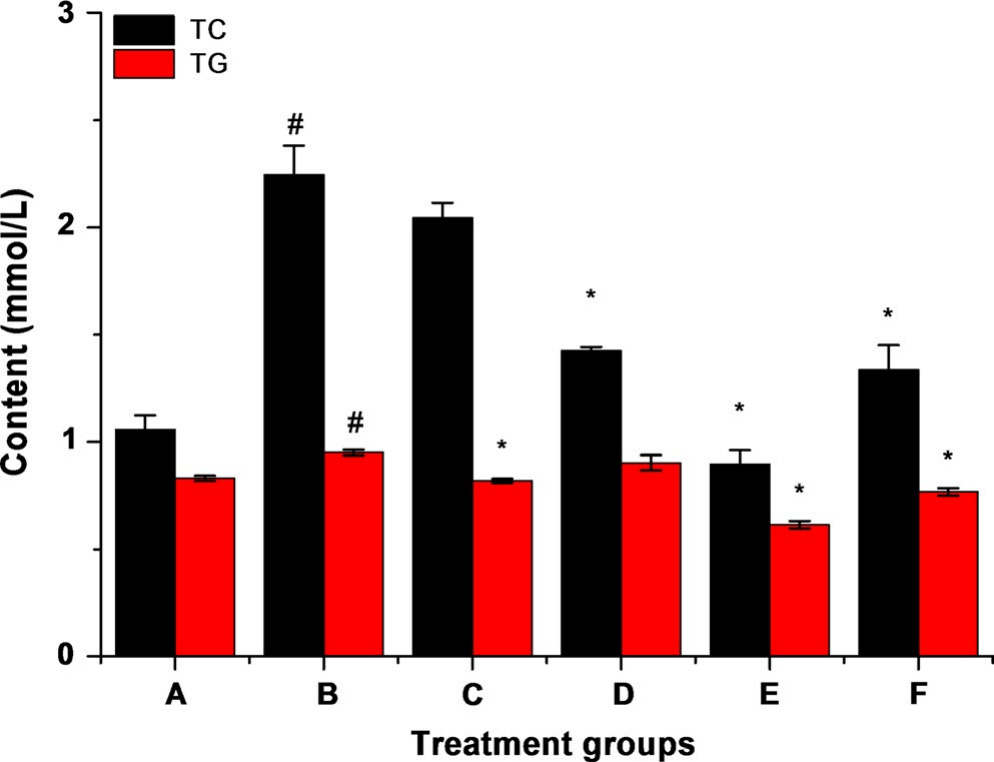
Effects of complexes on serum total triacylglycerol and total cholesterol levels. The data are presented as the mean ± *SD* (*n* ≥ 3), # means compared to control group (A), *p* < .05; * means compared to the alcohol group (B), *p* < .05

As shown in Figure 2, the levels of TG and TC in rat serum in the alcohol group (B) were significantly increased (*p* < .05) after liver injury induced by alcohol gavage, which were 1.2 times and 2 times the normal model group (A), indicating that alcohol gavage led to the liver lipid metabolism disorder. Compared with the group B, the decrease of TG in the LBP group (D) was not significant, and the decrease of TC in the ZnSO_4_ group (C) was not significant (*p *> .05). However, the reduction of TG and TC in group E and F had a significant difference (*p* < .05) correspondingly, and decreased by 25% and 60%, respectively.

### 3.3 Effect of LBP and ZnSO_4_ on serum transaminase levels in liver of rats

**Figure 3:**
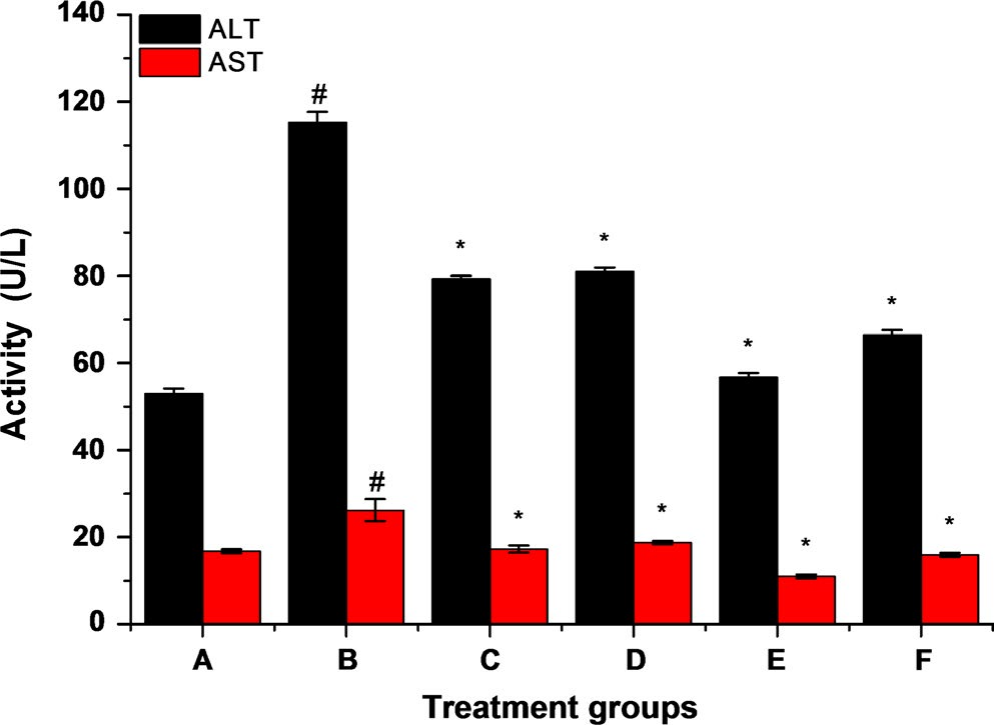
Effects of complexes on serum total triacylglycerol and total cholesterol levels. The data are presented as the mean ± *SD* (*n* ≥ 3), # means compared to control group (A), *p* < .05; * means compared to the alcohol group (B), *p* < .05

Oxidative stress is one of the important mechanisms leading to liver damage (Ana, Alma, Vázquez, Natalia, & Javier, 2016; Gao et al., 2017). Many investigations strongly suggest that liver damage produced by alcohol is mediated through oxidative stress (Albano, 2008; Ambade & Mandrekar, 2012). The elevation of serum transaminases can reflect the extent of liver cell damage and necrosis. As shown in Figure 3, compared with group A, the serum transaminase (ALT, AST) were significantly increased (*p* < .05) in group B, while compared with group B, they are all decreased significantly (*p* < .05) in the group C, D, E, F. It indicated that the group B was successful model of liver injury introduced by excessive alcohol intake, and LBP and ZnSO_4_ could reduce activity of serum transaminases.

### 3.4 Effect of LBP and ZnSO_4_ on antioxidant levels in liver of rats

As shown in Table 3, compared with group A, the SOD, CAT, GSH‐Px, GSH levels were significantly decreased (*p* < .05) in group B, but compared with group B, these antioxidant indicators in the group C, E and F were increased significantly (*p* < .05)inversely. The results show that the effect of LBP and Zn on oxidative damage of alcoholic liver were different, the synergistic effect of LBP and Zn is not a simple dose‐relationship.

**Table 3:** Effect of LBP and ZnSO_4_ on antioxidant levels and lipid peroxidation levels in rat liver

Groups	SOD (U/mg protein)	CAT (U/mg protein)	GSH‐Px (U/mg protein)	GSH (nmol/mg protein)
A	80.11 ± 0.67	81.99 ± 0.28	150.00 ± 1.59	6.81 ± 0.13
B	49.34 ± 5.81^#^	52.46 ± 0.03^#^	118.08 ± 6.45^#^	4.09 ± 0.39^#^
C	79.63 ± 5.05*	58.00 ± 0.48	145.14 ± 2.20*	6.02 ± 0.10*
D	72.37 ± 3.56*	40.24 ± 0.68*	142.06 ± 10.10	3.87 ± 0.14
E	98.44 ± 1.52*	68.66 ± 0.46*	169.61 ± 8.91*	9.12 ± 0.03*
F	92.01 ± 1.04*	56.22 ± 0.30	135.82 ± 4.15	6.45 ± 0.39*

Note

The data were expressed as the mean ± *SD* (*n* ≥ 3)；#means compared to the control group (A), *p* < .05; * means compared to the alcohol group (B), *p* < .05.

### 3.5 Effect of LBP and ZnSO_4_ on serum inflammatory factor and lipid peroxidation in liver of rats

**Figure 4:**
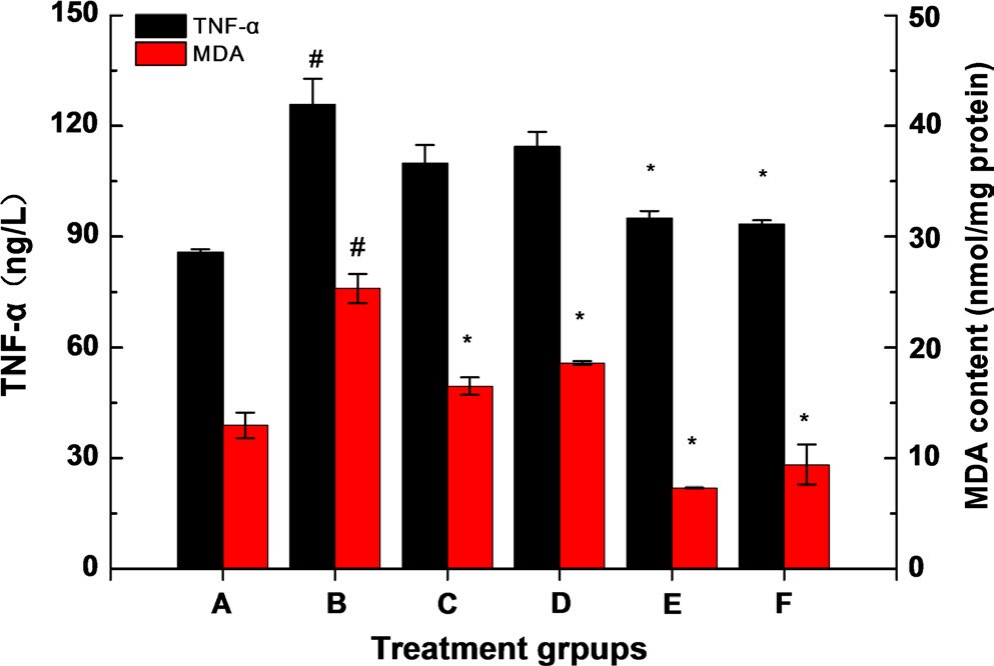
Effects of complexes on serum levels of TNF‐α. The data were expressed as the mean ± *SD* (*n* ≥ 3), # means compared to control group (A), *p* < .05;* means compared to the alcohol group (B), *p* < .05

As shown in Figure 4, compared with the group A, the TNF‐α and MDA level were increased significantly (*p* < .05) in group B. However, compared with the group B, the TNF‐α levels in the groups C and D showed no significant difference (*p *> .05), the groups E and F could significantly reduce the level of TNF‐α in serum (*p* < .05), while MDA level was decreased significantly (*p* < .05) in group C, D, E and F. The results showed that the combination of ZnSO_4_ and LBP had a synergistic effect on relieving lipid peroxide and chronic inflammation in rats.

### 3.6 Effect of ZnSO_4_/LBP on alcohol metabolism enzymes in liver of rats

**Figure 5:**
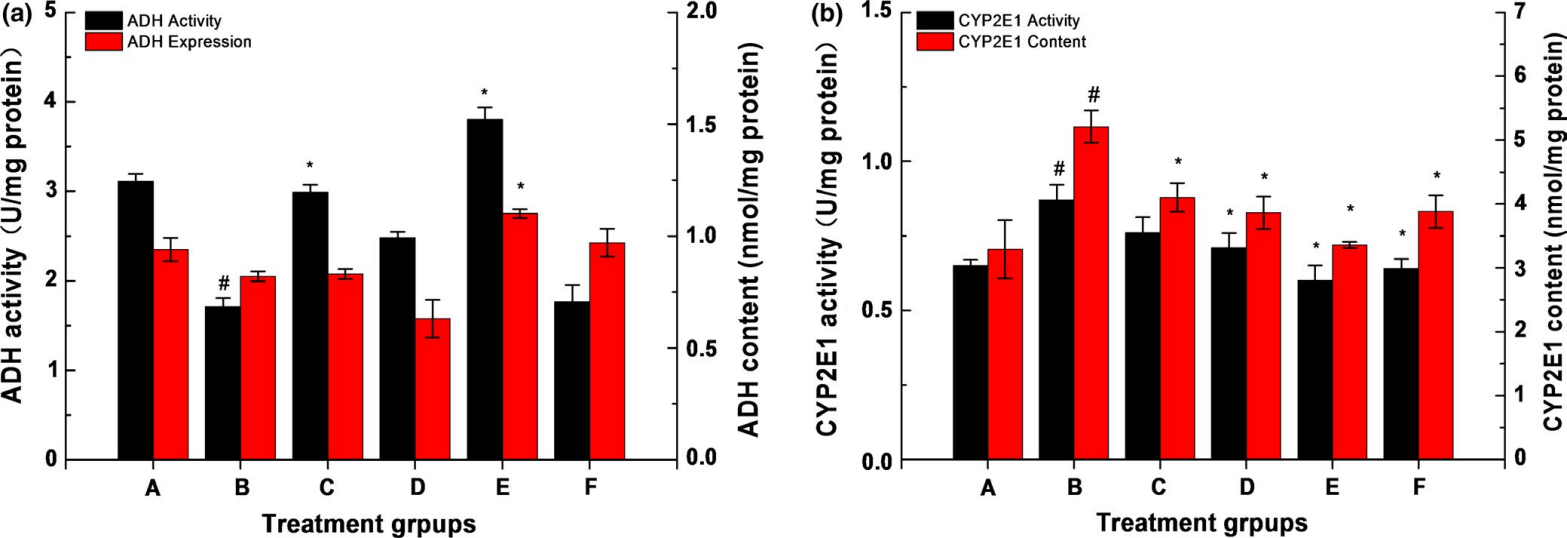
The effect of complexes on ADH activity and expression content in liver tissue (a). The effect of complexes on CYP2E1 activity and expression content in liver tissue (b). The data were expressed as the mean ± *SD* (*n* ≥ 3), # means compared to control group (A), *p* < .05; * means compared to the alcohol group (B), *p* < .05

As shown in Figure 5a, compared with the group A, the activity of ADH decreased significantly (*p* < .05) in group B, compared with the group B, groups were increased significantly (*p* < .05) in group C, D and E, while the content of ADH could be improved only in the group E (*p* < .05) compared with group B.

However, for the CYP2E1, as shown in Figure 5b, compared with the group A, both expression content and activity were significantly improved in group B (*p* < .05), which reduced in intervention groups (C, D, E, F) compared with the group B. Genetic polymorphism of enzymes involved in alcohol metabolism plays a relevant role in etiopathogenesis of alcohol disease and alcohol liver cirrhosis (Caro & Cederbaum, 2007; Cichoz, Partycka, Nesina, Celiński, & Saomka, 2006). The results showed that the complex of ZnSO_4_ and LBP could improve ADH activity to inhibit the production of acetaldehyde, on the contrary, reduce CYP2E1 activity, which could decrease alcohol‐derived reactive oxygen species (ROS).

### 3.7 Interaction between ZnSO_4_ and LBP on alcohol liver injury of rats

The pathophysiological process of alcohol‐induced liver injury is a complex process involving multiple factors. As shown in Table 4, in the group E, the CDI values of every index were ≤1, except CAT and GSH. While in the group F, the CDI values of ALT, AST, CAT, GSH were ≥1. These results showed that LBP and ZnSO_4_ have a synergistic effect on alcohol liver injury of rats, but was not a simple dose‐relationship.

**Table 4:** Different index of CDI in ZnSO_4_ and LBP complex groups

Groups	TC	TG	ALT	AST	MDA	TNF‐α	CYE activity	ADH activity	SOD	CAT	GSH‐Px	GSH
E	0.69	0.91	1.00	0.94	0.60	0.95	0.97	0.87	0.84	1.57	0.97	1.60
F	1.03	0.97	1.19	1.28	0.78	0.94	1.03	0.41	0.78	1.25	0.78	1.13

### 3.8 Histopathological analysis

**Figure 6:**
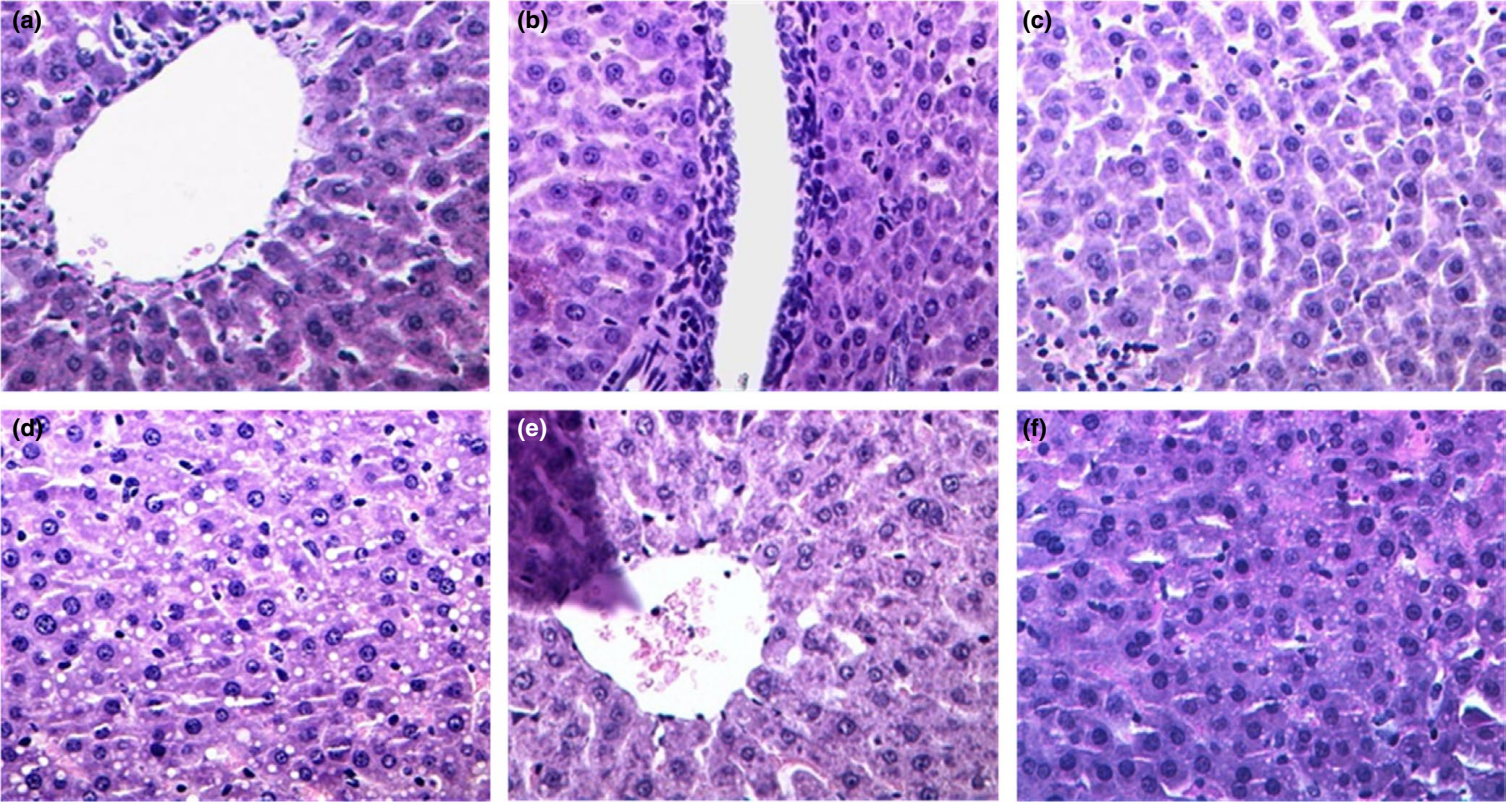
Hematoxylin and eosin staining of liver sections. The pathological changes of different groups of liver tissues in rats (HE, ×400), (a) normal group; (b) alcohol group; (c) ZnSO_4_ group; (d) LBP group; (e) low‐dose of ZnSO_4_ and LBP complex group; (d) high‐dose of ZnSO_4_ and LBP complex group

Liver biopsies are useful to evaluate the stage and severity of ALD. As shown in Figure 6, in the normal model group (A), the liver tissue structure of rats was complete and clear, the hepatic lobule structure is normal, the arrangement of the liver cells was a regular cord‐like shape, distributed radially around the central vein. Central venous endothelial was integrity, the liver cells were arranged in neat rows and closely, the cells structure was integrity, and the nuclei were large and round, showing deep staining of the nucleus and red staining of the cytoplasm. The structure of hepatic sinusoid was clear, and there was no inflammatory cell infiltration or change in the abnormality. The hepatocytes were polygonal, homogeneous in cytoplasm and cell nuclei were normal. No fatty degeneration, edema, necrosis or fibrous hyperplasia were found (Figure 6a). In the alcohol group (B), the normal structure of liver tissue was damaged, the hepatic cord was disarranged, the liver cells showed edema, and some hepatocytes were not stained with a deep nuclear structure, showing nuclear disintegration, cell necrosis or ballooning degeneration. Some vacuoles of different sizes can be seen clearly in the lobules of liver, and steatosis was extensive (Figure 6b). Compared with the group A, the liver tissues of both the low‐dose and high‐dose group of ZnSO_4_ and LBP complex were still slightly injured, but compared with the alcohol group, there no significantly inhibited alcohol‐induced liver injury (Figure 6e and f). Furthermore, the structure of liver cells and hepatic lobules is intact, the hepatic cord arrangement is regular, and there is only infiltration of a few inflammatory cells, most of the cells are normal, and the fat bubbles are significantly reduced.

## 4 DISCUSSION

Alcohol‐induced liver injury has an impact on the body's nutritional status (Campillo, Bories, Pornin, & Devanlay, 1997), and different alcohols have different impacts on body weight (Wang, Chen, Hu, Nan, & Chen, 2014). LBP exhibited the most significant treatment in reducing the body weight loss in rats, the body weight of LBP group was almost close to the normal model group, which could be related to the immune enhancement of LBP to the body (Zhang et al., 2014). However, there is no similar result in Daye Cheng's study (Cheng & Kong, 2011), the reason may be that animals of different genders and genotypes have different responses to the alcohol model (Gao et al., 2017).

It was showed that ZnSO_4_ and LBP can be more synergistic to repair a damaged lipid metabolism or reverse the lipid dysfunction caused by alcohol administration in rats and prevent alcohol‐induced fatty liver deteriorate in this study, which similar to their search that both LBP and Zn could reduce high fat‐induced liver damage and significantly reduce lipid accumulation (Cheng & Kong, 2011; Gan et al., 2018; Masci et al., 2018; McClain et al., 2017). Previous studies have shown that the mechanisms of ethanol impair oxidative balance within hepatic cells is complicated, autoimmune reactions associated with oxidative stress might contribute to fueling hepatic inflammation in ALD (Vidali, Stewart, & Albano, 2008); signaling intermediates regulated by oxidative stress that provokes proinflammatory responses in alcoholic liver disease (Ana et al., 2016);chronic ethanol‐associated alterations of mitochondria influenced the production of reactive oxygen and nitrogen species, which disrupted hepatic energy conservation in the chronic alcohol abuser (Bailey, 2003). The results of Table 2 showed that the effect of LBP and Zn on oxidative damage of alcoholic liver were different, the synergistic effect of LBP and Zn is not a simple dose‐relationship. Thus, the mechanisms of LBP and Zn on preventing oxidative damage of alcoholic liver should be research in‐depth in the future.

Genetic polymorphism of enzymes involved in alcohol metabolism plays a relevant role in etiopathogenesis of alcohol disease and alcohol liver cirrhosis (Caro & Cederbaum, 2007; Cichoz et al., 2006). It was shown that the complex of ZnSO_4_ and LBP could improve the activity and of ADH, reduces the activity and expression of CYP2E1, which indicated that a synergistic effects of ZnSO_4_ and LBP on the regulation of alcohol metabolism enzymes, although the details of the synergistic mechanisms were still unclear.

## 5 CONCLUSIONS

In conclusion, compared with the alcohol group, the complex of LBP and ZnSO_4_ could significantly decrease the levels of TG, TC, TNF‐α, MDA, ALT, AST and the activity of CYP2E1 in rats, which suffered chronic alcoholic liver injury, on the contrary, increased the activity of SOD, CAT, GSH‐PX, GSH and activity of ADH, effectively alleviate the liver tissue lesion. It was the combination of LBP and ZnSO_4_ that had a synergistic effect on the remission of alcoholic fatty liver in rats, but they are not a simple dose‐relationship. Ingestion of LBP and ZnSO_4_ could alleviate chronic alcoholic liver injury in rats by promoting lipid metabolism, inhibiting oxidative stress, controlling inflammatory responses, and regulating expression and activity of alcohol‐metabolizing enzymes. These results implied that LBP and zinc complexes may be applied for the treatment of ALD. However, clinical trials are needed to validate the beneficiary role of these supplements in patients with ALD, and further study is warranted.

## CONFLICT OF INTEREST

This study has not any potential sources of conflict of interest.

## ETHICAL APPROVAL

All animals were housed and cared for in accordance with the Chinese Pharmacological Society Guidelines for Animal Use.
